# Prevalence of Temporomandibular Disorders Between 2015-2021: A Literature Review

**DOI:** 10.7759/cureus.37028

**Published:** 2023-04-02

**Authors:** Ali H Alrizqi, Balsam M Aleissa

**Affiliations:** 1 General Dentistry, Ministry of Health, Bisha, SAU; 2 Dentistry, National Guard Health Affairs, Riyadh, SAU

**Keywords:** prevalence rate, global prevalence, local prevalence, temporomandibular joint (tmj) disorders, temporomandibular joint-tmj

## Abstract

The prevalence of temporomandibular disorder (TMD) is significantly high around the world. We conducted a literature review to determine the prevalence of TMD globally and in Saudi Arabia based on published studies. This review article collected 35 full-text articles after searching PubMed for TMD prevalence between 2015-2021. Assessing the prevalence of TMDs is important for several reasons, including providing an overview of the incidence of such disorders, educating the community, clarifying the gender and age group with the highest prevalence, establishing a program to prepare specialists to treat these disorders, and identifying the appropriate number of specialists by comparing TMD prevalence to Saudi Arabia's census. Out of 35 selected articles, thirty studies were done outside Saudi Arabia, and five were local. Less than 40% prevalence of TMD has been reported with associated factors such as gender, psychological status, and age. The female gender has shown a higher TMD rate than the male gender. Some authors have suggested conducting a temporomandibular joint (TMJ) assessment in the pediatric clinic. Moreover, TMD screening is an important tool for every patient visiting the dental clinic to assess TMJ status and treat TMD at early stages, especially in non-painful cases.

## Introduction and background

The temporomandibular joint (TMJ) is a diarthrodial joint situated between the condyle of the jaw and the mandibular fossa beneath the temporal bone, just in front of the external auditory canal [1]. Oral parafunction and temporomandibular disorders (TMD) are common issues in contemporary society. The muscles, occlusion, and periodontium are associated with the etiopathology of the TMJ [2]. The psycho-emotional element is shown to have a considerable influence in the literature, associated with the effects of other physical health-related variables such as systemic disorders, malocclusions, tooth loss, traumas, and microtrauma [3]. Stress, exhaustion, anxiety, despair, trouble sleeping, and a fast-paced lifestyle have a negative impact on the human psyche. Such people frequently perceive TMD to have a muscular component [2]. Multiple studies [3,4] have shown that TMD coexists with various other conditions, including fibromyalgia, back or spine pain, chronic fatigue syndrome, spastic colons, sleep problems, congenital malformations, migraines, and arthritis. The increased prevalence of TMD has been linked to physical, psychological, and hormonal changes throughout pubertal development [4].

The age of this group is another characteristic that predisposes to the development of masticatory system problems in students since symptoms peak between the ages of 20 and 40. Women of breeding age are the majority of TMD sufferers [5]. The prevalence of TMD increases globally throughout adolescence and may vary from 7% to 30% [5]. According to several investigators [5,6], women experience the signs of masticatory system abnormalities more often than men. This might be brought on by hormonal changes in biology and psychological variables. The researchers claim that students experience greater stress than the general population and are far more likely to get TMD and oral dysfunction [5]. Exams, presenting research papers, the need to become independent, financial difficulties, studying in an uncomfortable posture, and poor academic performance are only a few of the potential stressors.

The OPPERA (Orofacial Pain: Prospective Evaluation and Risk Assessment (OPPERA) ) investigations, which support a biopsychosocial model of disease and the complex etiology of TMD, have been published. In contrast, the importance of malocclusion, formerly thought to be one of the primary aetiological impacts of TMD signs and symptoms, has been downplayed. Crossbite, maximal intercuspal instability, and Class II malocclusion have all been linked to increased probabilities of TMD [5]. Moreover, those with a history of TMD may be more likely to have TMD symptoms due to occlusal alterations. This leads to a complex cause-and-effect correlation between TMD and malocclusion [4,5].

The importance of a healthy TMJ cannot be overstated. TMDs are the cause of orofacial pain. TMDs are a constellation of signs and symptoms related to the TMJ system that can be diagnosed by clinical examination, such as jaw movement limitations, sounds (clicking and crepitation), pain from jaw movement, and pain on masticatory muscle palpation. Multiple factors may cause TMDs, including occlusal interferences, neoplastic growth, disharmony between the condylar head and temporal fossa, destructive (nonfunctional) movement of the mandible (e.g., bruxism), emotional stress, malposition or loss of teeth, and teeth clenching or grinding habits [7,8].

In 1992, Dworkin and LeResche introduced Research Diagnostic Criteria for Temporomandibular Disorders (RDC/TMD) for clinical research as a standard to increase the consistency of studies evaluating TMDs in different global regions [5]. The RDC/TMD protocol consists of two axes. Axis I deals with the physical properties of TMD, while axis II deals with the psychological components. In 2014, TMD experts agreed to establish an expert-based Diagnostic Criterion for Temporomandibular Disorders (DC/TMD). This assessment protocol can be used in a clinical setting for research and assessing patients before treatment [7].

Assessing the prevalence of TMDs is important for several reasons, including providing an overview of the incidence of such disorders, educating the community, clarifying the gender and age group with the highest prevalence, establishing a program to prepare specialists to treat these disorders, and identifying the appropriate number of specialists by comparing TMD prevalence to Saudi Arabia's census.

There is a significant need to know the average prevalence of TMD after implementing the new DC/TMD assessment protocol so Saudi national authorities can plan for the resources TMD patients need. This literature review will assess global TMD prevalence based on studies published between 2015 and 2021.

## Review

Literature review

Out of 35 selected articles, thirty studies were done outside Saudi Arabia, and five were local.

Search strategy 

The search approach included seeking trustworthy sources to provide information that elaborated on the subject. In order to find a range of papers that provided data from various angles depending on the area of concentration for each research, numerous databases were employed. Creating keywords that may be utilised to focus on a particular piece of content was the first step in the search strategy. "Temporomandibular joint" and "Temporomandibular dysfunction" are among the keywords. EBSCO Host, PubMed, SCOPUS, and Google Scholar are the databases used.

Inclusion and exclusion criteria 

The inclusion criteria for publications for this systematic review involve establishing specific criteria that must be satisfied before the research is chosen. Finding documents that concentrate on the prevalence of TMD was the first criteria. Secondly, all academic, peer-reviewed papers were taken into consideration for review. All of the chosen pieces were also published in English. The study's emphasis on TMD prevalence was determined by reading the abstract, which was the first requirement for exclusion.

Bias risk assessment 

The Cochrane risk of bias assessment technique was used to ascertain if any kind of bias existed in the chosen studies. By focusing on several components of reporting, behaviour, and trial design, this tool is essential in determining the risk of bias in randomised trials. Using the Cochrane risk of bias assessment tool is justified in order to examine the validity of the results by looking at the methodology of the study. Performance, selection, reporting, attrition, and other bias make up the five domains of the tool .

Literature review (global)

In men, TMD prevalence varied from 10.6% to 68.1%. A prevalence of 21.2% to 72.4% was observed in females. Findings in all research reported that women were more likely to develop TMD in comparison with men.

Young girls aged 8, 14, and 18 years old did not exhibit any significant differences in TMD prevalence, according to Aboubakr and Elkwatehy [9]. In contrast, additional investigations found that adult patients had a greater prevalence of TMD than children (18 years old and below). Patients above 20 had considerably more TMJ tones as detected by Lai et al. [10]; they observed that TMD prevalence steadily declined beyond the age of 40 [9].

In a research from Saudi Arabia [10], a dental classification was employed to identify malocclusion, skeletal connection, the Index of Orthodontic Treatment Need (IOTN), and occlusal traits were used. Lövgren et al. [11] concluded that there was no connection between TMD and dental classification. IOTN and TMD symptoms or indicators were not significantly correlated. Nonetheless, the researchers that looked into occlusal characteristics concluded that open bite, deep bite, and posterior crossbite seemed to be linked to TMD. They observed that higher overbite and a larger interincisal angle were related to TMJ sounds. Alahmary et al., on the other hand, discovered no conclusive link between joint noises and functional occlusion [12]. 

According to the DC/TMD, the most common TMD symptom was myalgia, which had a frequency of 30%. Those who were diagnosed with TMD pain had substantially higher Graded Chronic Pain Scale (GCPS) classifications than people who were not diagnosed with TMD pain (p=14.001, two-sided). People in total (65%) reported experiencing local, regional, or broad discomfort. Regarding the degree of depression, anxiety, stress, or pain catastrophizing, no significant differences existed between those with any TMD and those without TMD. Somatic awareness varied significantly among people with any TMD. Also, compared to those without TMD, those with any TMD had substantially higher scores for oral parafunction and functional jaw restrictions [13]. Compared to the frequency in the general population (10%), previous research has shown that TMD is prevalent among dentistry students at a rate of 30%-50% [14]. In contrast to prevalence based only on self-reported symptoms, prevalence based on clinical exams following established diagnoses and trustworthy and valid questionnaires is likely to vary [15].

The research comprised 754 students who completed the survey and the form for the clinical evaluation; 354 boys and 400 girls, all were 19 years of age. TMD was present in 31.7% of the population with no discernible gender difference. Although headache and locking had a low prevalence of 13.8% and 33.9%, respectively, orofacial discomfort and joint noise were the most prevalent symptoms of TMD. The patients were then separated into three groups, each having a prevalence of 15.5%, 66.9%, and 17.6% for pain-related TMD, intra-articular TMD, and combination TMD [16].

Compared to students without TMD, individuals with TMD exhibited a higher incidence of empty chewing, unilateral chewing, sleep bruxism, and awake bruxism [17]. Regarding occlusions, it was shown that students with TMD had a considerably greater incidence of anterior teeth overbite and anterior teeth overjet [17]. Regarding the psychological component, individuals with TMD showed a considerably greater incidence of sadness, anxiety, and sleep disruption [18].

Among the 70-year-olds [19], there was no discernible gender difference, but among the 80-year-old individuals, there was a greater percentage of women (p = 0.001). Data cross-sectional in the group of people aged 70 and older, 12% of the women and 7% of the males reported having some, quite significant, or severe discomfort in the TMJ areas (p =0.001). Women and men aged 80 had a matching prevalence of 8% and 7%, respectively (p > 0.05). In the 70-year-old group, the prevalence of issues with TMJ noises and difficulty extending the jaw was similarly considerably greater in women than in males (p 0.001), but there were no significant gender differences in the 80-year-old group (p > 0.05). In terms of bruxism, there were the biggest variations across the age and gender categories. For instance, compared to the 80-year-old males, the 70-year-old women reported twice as many bruxism difficulties (21%). For all symptoms, the incidence of quite significant and serious issues was less than 3%, with the exception of bruxism in women over the age of 70 (4%) [15].Despite the fact that more men than females presented with TMD, there was no discernible difference in the cross-tabulation between gender and TMD. The younger subjects seemed to have a larger prevalence of TMD based on age but the correlation was not statically important. There was a strong correlation between education and TMD; participants with lower levels of education had more TMD symptoms than those with higher levels of education (p=0.002). The administrative personnel had a greater incidence of TMD than academic staff, and the association between the two was shown to be statistically significant (p-value 0.000) [19].

In the United Arab Emirates (UAE) [20], a study evaluated the prevalence of TMD among patients who visited dental clinics at the Ajman University of Science and Technology. One hundred sample subjects were evaluated using the RDC/TMD criteria. AlShaban et al. found that more than 40 participants (> 40%) exhibited TMD. Surprisingly, younger and male patients (19-29 years old) had a greater prevalence of TMD than the older age group (60-69 years old) and female patients [21].

A study was published in 2018 that assessed the prevalence of TMD among the Nepalese population [22]. The authors administered the Fonseca anamnestic and Fonseca questionnaires to the sample which consisted of 500 participants with a mean age of 20 [23]. Rokaya et al. found that more than 25% of the examined sample had mild TMJ problems, while only 3% of the participants had moderate TMJ problems [24]. An Indian study [25] was published in 2018 that assessed the prevalence and severity of TMD among orthodontic patients visiting a dental college. Three hundred ninety patients were divided into two groups based on their ages (12-18 or 19-30). The participants were given Fonseca’s questionnaire, and it was found that in the 12-18 age group, 18% of males and 12% of females had TMD [25].

On the other hand, 30% of females and 19% of males in the 19-30 age group revealed the presence of TMD. Jain et al. reported that the prevalence of TMD was higher in the 19-30 age group than in the 12-18 age group [26]. In 2018, Bertoli conducted a study to evaluate the prevalence of TMD among 934 Brazilian participants from 10-14 years old. The author used the DC/TMD protocol in this study to diagnose TMJ. The author found that over a third of the examined sample was diagnosed with TMJ. The most common symptom was myofascial pain, headache, neck pain, and TMJ sounds. In addition, the author discovered that the incidence of TMD in females was significantly higher than in males. Bertoli advised conducting TMJ assessments in pediatric dental clinics [27].

In 2020, a study was conducted in Brazil to evaluate the prevalence of TMD, parafunctional habits, depression, and anxiety in COVID-19 social isolation among students and the associations between these variables. The authors found a positive correlation between COVID-19 social isolation and the prevalence of TMD. de Melo Júnior et al. also identified a relationship between parafunctional habits/oral behaviors and the incidence of TMD [28]. A study published in 2020 aimed to assess the prevalence of TMD among the Finnish adult population; 1,557 participants aged 18 or older were involved in the study. Qvintus et al. found that more than a third of the study sample had at least one sign of TMD, and less than 10% suffered from pain symptoms. The authors also found that TMD prevalence increased with low general health, female gender, and low educational levels. Thus, the author recommended conducting a general health assessment of patients seeking TMD treatment [29].

Graue et al. [30] reported in their research that disc displacement was the first and most common cause with a frequency of 5.4% (9/167). Myalgia was the second most common cause of TMD with a frequency of 3.0% (5/167). Three participants (1.8%) had more than one TMD diagnosis. In two individuals (1.2%), there was a combination of myalgia and arthralgia; in one participant (0.6%), there was a combination of arthralgia and headache. One participant (0.6%) had disc displacement with intermittent locking. Twelve (60.0%) of the 20 individuals diagnosed with TMD in the last 30 days had discomfort in their jaw, temple, or, alternately, the ear in front of their ear. Among them, 10 participants (eight females) said their discomfort was worse when they chewed, opened their mouths, spoke, or chewed. On the DC/TMD diagnostic survey, nine out of the 20 subjects (45.0%) with a DC/TMD diagnosis reported having temple headaches. On the DC/TMD questionnaire, 29 out of 147 individuals (19.7%) who had no DC/TMD diagnosis indicated that they had had a headache over the previous 30 days. After the DC/TMD protocol conducted the clinical assessment, none of the research participants reported experiencing any negative effects [30].

Another study assessed the prevalence of TMD among the Australian population. An online survey was distributed to all participants. Lung et al. found that more than two-thirds of the participants had TMD. In addition, they found that females had TMD more often than males. TMJ pain was the most common symptom among the study subjects [31]. In 2019, Alrashdan et al. surveyed 368 adults in northern Jordan to assess the prevalence of TMD using the DC/TMD protocol (axis I). The authors found that 98 adults (26.6%) in their tested sample had one TMD symptom, and over 70 patients (19%) suffered from pain related to TMD. Once again, females exhibited a higher rate of this diagnosis than males [32].

In 2019, Lai et al. performed a systematic review to evaluate the prevalence of TMD and define the association between TMD and age, gender, and malocclusion. Lai and coauthors mentioned that according to a systematic review, TMD prevalence varied between studies, ranging from 21.1% to 73.3% [10]. Lai et al. also found that the prevalence of TMD was higher in age groups above 18 years than in younger age groups. On the other hand, there was no significant correlation between malocclusion and TMD incidence [23]. A Brazilian study published in 2019 by de Melo Júnior et al. [28], evaluated the prevalence of TMD and chronic pain levels among Brazilian adolescents. A total of 1,342 subjects were examined using the RDC/TMD criteria to evaluate the presence of chronic pain and its severity level. TMD was associated statistically significantly with female gender (p = 0.017), headache/migraine in the last six months (p= 0.001), chronic pain (p =0.001), and severity of chronic pain (p =0.001). In the final model, logistic regression revealed a correlation between the existence of TMD and the frequency of recent headaches and migraines [10].

In addition, variables such as female gender, presence of chronic pain, level of chronic pain, and headache/migraine within the last six months were associated with TMD. On the other hand, factors such as age and economic class did not affect the presence of TMD [33]. Another study evaluated the prevalence of TMD among the adult Italian population. A total of 4,299 adults were involved in this study, and a questionnaire was distributed. Iodice et al. found that 30% of the study subjects reported TMJ clicking, and oral parafunctional habits were identified in 92% of the study subjects [34]. 

TMD and psychological factors

In 2020, a study evaluated the prevalence of TMD and anxiety levels among ballet dancers. Christidis et al. administered a questionnaire to evaluate anxiety status and clinical examinations to identify TMJ. The most common symptoms among the study subjects were arthralgia and disk displacement with reduction. However, the authors found no correlation between anxiety levels and the presence of TMD [35]. In 2018, a study evaluated the prevalence of TMD among college students in Brazil. The author intended to assess the correlation between the prevalence of TMD and other factors, such as emotional stress, parafunctional habits, and gender. Moreover, the author wanted to determine whether there was a correlation with oral health-related quality of life (OHRQL) [36]. A total of 303 students were invited to participate voluntarily in the study. Paulino et al. found that TMD was more highly associated with the female gender and participants experiencing stress. In addition, they found a relationship between TMD and parafunctional habits. Participants with TMD also had poor OHRQL. The authors recommended educating students and teachers about the importance of the early diagnosis of TMD [37].

A Lebanese study was published in 2020 that evaluated the prevalence of TMD and its association with psychological factors, such as stress, anxiety, and depression, among the Lebanese population [38]. Kmeid et al. collected their sample subjects from two different zones; 459 people from all over Lebanon participated in the study, and the other 37 came from a specific otolaryngologist clinic. The authors found that almost one-tenth of the Lebanese population had TMD, and more than half of these cases were female. TMD was much more common in people who had been to otolaryngologist clinics than in the general population. Moreover, people with high stress, anxiety, and depression had higher TMD severity [38]. In addition, the authors found high stress, depression, anxiety, and TMD scores in the patients visiting the otolaryngologist clinic compared with the general Lebanese population [28]. In 2020, a study evaluated the prevalence of TMD among the North Indian population. Chaurasia and coauthors assessed 1,009 patients using the RDC/TMD protocol. They found that the most common symptom among the study subjects was TMJ clicking. In addition, patients between 18-35 years old had the highest prevalence of TMD [39]. A recent study was conducted in Poland in 2021 to evaluate the prevalence of TMD among patients visiting prosthodontic clinics at Jagiellonian University; 344 patients (210 females and 124 males) were examined for TMJ function, aged 41-68. Less than half of the examined patients were pain-free, and 173 (50.3%) suffered from painful TMD. The author found that most painful cases were related to masticatory muscle problems, and most of the evaluated painful cases were female [40].

Literature review (local)

The first research that examined TMD prevalence in the adult population of Jeddah, Kingdom of Saudi Arabia. It was drawn from the city's many geographic regions to ensure that the sample was representative. In Jeddah, 35% of the adult population had TMD. This is a somewhat high number, but it is consistent with previous research in various demographic subgroups of the Kingdom of Saudi Arabia. Zwiri and Al-Omiri found that 49.7% of North Saudi University students had at least one sign or symptom of TMD. They said it was more prevalent in women, consistent with other results [41-43].

In the Kingdom of Saudi Arabia's Jeddah, Al-Khotani et al. [44] conducted a cross-sectional study of children and teenagers enrolled in schools. After using the TMD/pain screener, they performed an RDC/TMD-based evaluation. They said that 27.2% of the population had been given at least one TMD symptom. To determine the frequency and severity of TMD in female dentistry students in Riyadh, Kingdom of Saudi Arabia, AlHussaini et al. [45] employed an electronic questionnaire. They assessed the amount of anxiety using the Fonseca Anamnestic Index (FAI) and the Zung Anxiety Self-Assessment Scale (SAS). They claimed that 62.8% of their sample had high anxiety levels linked to TMD. The gender selection in their study's investigated population may account for the greater occurrence rate [45].

Four hundred edentulous patients were assessed by Alzarea et al. when they were in Skaka, Kingdom of Saudi Arabia's Al-Jouf dentistry school. They stated that 60.5% of the patients in this group had TMD. The high occurrence rate in their results may be explained by occlusal instability [46].

According to Nadersha et al. [47] findings, women were substantially more likely than men to have TMD (p = 0.0008). This agrees with a lot of earlier investigations. The precise cause of this gender disparity is not investigated yet. Different types of joint collagen in the TMJ retro-diskal zone, laxity of the joint ligaments, hormonal variables, and increased intra-articular pressure are some potential causes. An analysis of the gender differences in TMD prevalence in adults was done by Hilgenberg-Sydney et al. They chose five publications with a total of 2518 patients for the meta-analysis. They found that women had a two times higher chance of acquiring TMD [42].

A recent study was published in 2021 that assessed the prevalence of TMD among dental clinic patients at King Faisal University in Jeddah. Alogaibi et al. identified a relationship between the ages of the patients and TMD, as the prevalence increased with patient age. In addition, the authors found that females had a higher TMD prevalence than males [48].

Discussion

This literature review aimed to assess the prevalence of TMD globally and locally since 2015, as the new DC/TMD protocol was released in 2014 [6]. According to this review, the prevalence of TMD in most studies ranged from 30%-50%. However, some studies found a prevalence out of this range, either higher or lower. A Brazilian study that evaluated TMD prevalence among preparatory school students showed a very high prevalence (89.8%) of TMD. This high percentage might have been due to the habits of the participants recruited for the study, as most (95.4%) had parafunctional habits [26]. Another study in India assessed TMD prevalence among health and science students, finding the disease in 97% of the study sample; this is a very high rate compared with other studies evaluating TMD prevalence [36]. This high prevalence of TMD might have been due to stress among college students, which may lead to an increase in the incidence of TMD.

Generally, females exhibit a greater prevalence of TMD than males. The exact reason behind this remains unknown. However, United Arab Emirates studies have indicated that males have a higher TMD prevalence than females, which might indicate that ethnicity influences TMD prevalence [16].

A Spanish study mentioned that the rural population had a higher TMD prevalence than urban residents, which is the opposite of what a Norwegian study found [37-39]. Urban life can be stressful and busy due to multiple factors, such as hectic road traffic and exhausting work, which lead to distress. On the other hand, rural life is often peaceful, relaxed, and non-stressful, which contributes to decreasing the incidence of TMD.

We can conclude that the widespread rate of TMD worldwide is an issue that should be considered and dealt with. In addition, community education and awareness concerning the importance of TMJ screening and the risks of TMD to general health should be considered. Moreover, delays in TMD management may generate higher costs for the government and individuals since they must treat a patient in multiple specialized areas, which would be reflected negatively on the waiting list for that specialty. In addition, a patient not aware of TMD may not seek treatment, which could lead to the deterioration of TMJ's health status and aggravation of the problem.

The General Authority of Statistics is responsible for the Saudi Arabia census (Table 1) [40]. If an average of eight patients see a TMJ specialist daily, 40 patients can be seen per week. In addition, patients involved in TMD clinics need more than six months to be managed properly and finish their treatment course. This reflects the high demand for specialized treatment in Saudi Arabia to check, evaluate, treat TMJ, and educate the Saudi population about TMD. This could help avoid the formation of TMD and prevent the TMD rate from worsening. According to the local literature review, an average of 40.8% of the Saudi population has TMD out of eight million citizens. Thus, we need at least 8,000 TMJ specialists to fulfill the need for this specialty.

**Table 1:** Population in Saudi Arabia

Age Group	Total
5-9	2,262,377
10-14	2,076,011
15-19	1,846,634
20-24	2,072,516
25-29	2,068,030
30-34	1,877,577
35-39	1,677,696
40-44	1,357,837
45-49	1,153,834
50-54	925,199
55-59	727,311
60-64	504,441
65+	952,811
Total	21,690,648

Moreover, there should be a collaboration between different specialties and TMJ clinics to accelerate the management of TMD cases related to psychological disorders, such as depression, so that these patients can be transferred from dental departments to psychiatric clinics as needed. Moreover, TMJ clinics can shorten the waiting lists for other specialties by preventing TMD cases from getting worse. For example, patients with clenching might end up with crowns and increases in the vertical height of occlusions if the problem is not treated and managed early [48].

## Conclusions

In conclusion, the prevalence of TMD is high (30%-50%) worldwide. Female gender and psychological status are associated with TMD. Adolescents can suffer from TMD, so the DC/TMD assessment protocol is advisable for children. Global authorities need to prepare the required resources to serve TMD patients, and dental schools should develop academic programs with clinical training in the field of orofacial pain and TMD.
